# The world’s first mobile lymphoedema unit: Wales leads the way

**DOI:** 10.3332/ecancer.2013.ed25

**Published:** 2013-09-03

**Authors:** Rachel Iredale, Richard Pugh, Melanie Thomas

**Affiliations:** 1 Tenovus, Cardiff, Wales; 2 Singleton Hospital, Swansea, Wales

Wales is leading the way in the development of mobile lymphoedema assessment and treatments. Lymphoedema, a chronic swelling due primarily to a failure of lymph drainage, is an incurable condition requiring lifelong management. Lymphoedema can be caused by a variety of conditions including obesity, venous disease, and recurrent cellulitis. Cancer treatment, particularly radiotherapy or surgery, is also a recognised cause of lymphoedema (DiSipio *et al* 2013). Patients can remain at risk of developing lymphoedema for some time after their treatment, yet it is a problem that is poorly recognised by healthcare professionals (Moffatt *et al* 2003).

Lymphoedema is well documented as being an extremely debilitating condition with a profound impact on patients’ quality of life (Kwan *et al* 2002). Symptoms include gross swelling, pain, and mobility problems, and patients often experience difficultly performing activities of daily living (Morgan *et al* 2005) In addition, the psychological impact of lymphoedema on patients is considerable (Meiklejohn *et al* 2013).

The Welsh Government has developed a ‘*Strategy for Lymphoedema*’ acknowledging that improving patient access to specialised lymphoedema services would be crucial as specialist services are highly successful in controlling swelling and associated symptoms (Welsh Assembly Government, 2009). There was wide variation in the organisation and delivery of lymphoedema services across the seven Health Boards of Wales; some Health Boards had dedicated services delivered by a range of allied healthcare professionals, others had nothing and the diversity of provision was not driven by patient need.

To improve patient access to lymphoedema services, Tenovus, Wales’ leading cancer charity, working in partnership with NHS Wales, launched a three month pilot mobile lymphoedema clinic on board the Tenovus Mobile Cancer Support Unit in 2012. Initially the Mobile Unit was developed to provide chemotherapy treatment and associated psychosocial support services to patients in a local setting, reducing journey times and providing a welcoming and familiar environment for patients and their families. An early evaluation of the Mobile Unit showed high levels of patient satisfaction, both with the environment itself and the level of support provided (Iredale *et al* 2011).

For the pilot lymphoedema project the Mobile Unit was staffed by specialist practitioners from NHS Wales able to provide multidisciplinary care to lymphoedema patients. Developed with reference to the International Lymphoedema Framework (2006), this model of care included skin care, multilayer lymphoedema bandaging, lymphatic drainage, compression garment fitting, and advice on self-management techniques. This approach was designed both to manage patient’s symptoms and improve their quality of life, giving them the support and education they need to manage their condition at home.

Using mixed methods we explored whether a mobile setting is a viable way of assessing, treating and educating people with lymphoedema and investigated the attitudes that patients and staff had about the Mobile Unit. A total of 14 lymphoedema clinics were held on the Mobile Unit with 64 patients treated over a three month period; most patients had either breast, colorectal or neck cancer. Out of 57 respondents 37 patients (65%) stated that they had previously received treatment for their lymphoedema in a hospital setting. When asked how their treatment on the Tenovus Mobile Unit compared with their previous appointment, no patients stated that it was *worse* than their previous appointment with the majority (n=22, 59%) saying that their treatment on the Mobile Unit was *better* than their previous treatment. The most cited reasons for why the Mobile Unit appointment was better than a previous appointment related to the convenience of receiving treatment closer-to-home, which reduces travelling time and cost. The average distance travelled per journey, per patient to the Mobile Unit was 10.8 miles compared to 21.4 miles for a previous appointment. On average, our sample of lymphoedema patients spent 31 minutes travelling to the Mobile Unit compared to an average of 55 minutes to a usual appointment. All patients held extremely positive attitudes towards the Mobile Unit rating highly the convenience of using the Unit, its general environment, the quality of service and care provided, and its comfort and cleanliness.

Mobile Units provide practical, patient-centred solutions to meeting the needs of cancer patients. The Welsh Government’s current strategy for cancer, *Together for Health, the Cancer Delivery Plan for the NHS up to 2016*, was published in June 2012 (Welsh Government, 2012). The NHS in Wales currently spends about 7% of its budget on cancer services. In 2010–2011 this amounted to just over £347 million. The Cancer Delivery Plan clearly demonstrates the need for cancer services to be delivered outside of the hospital setting and as close to home as possible, whilst maximising patient preferences. Established Welsh clinics have reported a substantial increase in the numbers of lymphoedema cases in the last 5 years.

With that in mind Tenovus are now building a bespoke Mobile Unit exclusively for lymphoedema patients; a custom built trailer with fully functioning treatment facilities and counselling rooms. We will be working in partnership with all seven Health Boards in Wales to see at least 25 patients per day for lymphoedema treatments and even more for group assessments, as well as offering education and support groups around cancer rehabilitation that will operate after hours. All of the services provided on the Tenovus Lymphoedema Mobile Unit will be free of charge at the point of use for anyone affected by cancer in Wales.

Watch Rachel Iredale discuss the unit with Prof Gordon McVie.
